# Analysis of Selected Organophosphorus Compounds and Nano-Additives on Thermal, Smoke Properties and Quantities of CO and CO_2_ of Epoxy Materials

**DOI:** 10.3390/ma16093369

**Published:** 2023-04-25

**Authors:** Sebastian Staszko, Marzena Półka

**Affiliations:** Faculty of Safety Engineering and Civil Protection, The Main School of Fire Service, 52/54 Slowackiego Street, 01-629 Warsaw, Poland

**Keywords:** flame retardants, fire safety, nano-additives, toxic products

## Abstract

Majority of anthropogenic air pollutants enter the atmosphere as a result of material combustion, industrial production and transport. Fires not only cause air pollution, but also disrupt ecosystems. Knowledge of the flammability parameters and proper flame-retardant modification of materials hinders the origin and spread of a fire, while also protecting against air pollution. The aim of this study was to obtain fire-retardant modifications of the epoxy resin, and then to analyse the effect of the introduced additives on the rate of heat release, the thermokinetic properties and the toxicity of volatile combustible products. The modifiers of the epoxy resin were organophosphorus compounds and aluminium and magnesium hydroxides, with a grain size of 10 nm. The introduced additives were found to be effective flame retardants as they reduced the rate of heat release and the amounts of toxic products of thermal decomposition and combustion. The HRR_max_ and HRR_av_ values of all fire-retardant modifications were lower compared to the corresponding HRR values of the unmodified epoxy material.

## 1. Introduction

Negative environmental impacts of fires range from adverse to inappropriate, depending on their extent. These impacts can be divided into three categories: immediate (fire brigade intervention), long-term (months and years after the fire) and inapplicable. The initial assessment considers the atmosphere and corresponds to a classification of the severity of the impacts based on the different phases of the fire. Polymeric materials such as cured epoxy resins are often found in construction, transport, electronics and electrical engineering, medicine and households. Very good mechanical resistance, high chemical resistance, moisture resistance, ease of processing and colouring makes them suitable for a wide range of applications [1,2,3,4]. Unfortunately, their principal drawback in practical application is their flammability and the high intensity of heat and smoke released during the combustion process. For this reason, the aim of this study was to attempt an effective flame-retardant modification of these polymers and to carry out an analysis of the heat release rate, thermogravimetric analysis, an evaluation of the toxicity of thermal decomposition and combustion products and determination of the mechanical properties.

To date, halogen compounds have been effective and popular flame retardants for epoxies. Regrettably, their use is limited or banned due to the toxic effects of thermal decomposition and combustion products on humans and the environment, because of the release of large quantities of irritant and toxic gaseous substances during combustion, including bromine, hydrogen chloride, dibenzodioxins and dibenzofurans [5,6]. These limitations have resulted in a surge of interest in halogen-free combustion reaction inhibitors for polymeric materials that reduce the rate of heat release from modified materials and limit the emission of toxic thermal decomposition and combustion products [7]. Confirmation of this line of research is provided by the statistics of the State Fire Service Headquarters for the year 2020 [8], which shows that 360 people have died in residential fires in Poland, and as other statistics show [9], most of them became victims after inhaling smoke and toxic combustion products from, among other things, plastics. An analysis of the rate of heat release modification of fire-retardant epoxy materials included in interior finishes and furnishings is, therefore, important in the study of fire environment hazards. Fire retardants containing phosphorus and its appropriate derivatives have been widely used in polymers, which reduce flammability due to their high reactivity, high thermal stability and excellent flame-retardant properties [10,11,12,13,14]. They are pro-ecological and can be effectively used as replacements for halogen flame retardants, as they usually produce relatively little smoke during the combustion of the modified polymeric material, creating a stable carbon layer, and the products of thermal decomposition and combustion are moderately toxic [3,15,16]. The carbonised layer can prevent further thermal decomposition of the polymer material, as well as inhibit the diffusion of combustion products to the gaseous phase formed after thermal decomposition [17]. Phosphorus-based flame retardants can be divided into inorganic phosphorus and organophosphorus compounds. Inorganic phosphorus flame retardants refer mainly to red phosphorus [18], ammonium polyphosphate [19], etc. Organophosphorus flame retardants include, e.g., phosphate esters [20] and phosphorus-containing polyols [21].

Currently, one of the most effective methods of fire-retardant modification of polymers is the introduction of nano-additives to the polymer matrix [22]. The nanoparticles are finely dispersed in the polymer matrix so that most of the polymer is in the interfacial polymer. In the literature, you can find many review articles and compact publications on the uniqueness of nanocomposites as additives to polymers to reduce their flammability [23,24,25,26]. Nano-additives act in the condensed phase, slowing down (but not stopping) polymer mass loss under fire conditions by creating a nanoparticle-rich fire barrier [27,28,29]. This reduces the maximum rate of heat release and inhibits the polymer softening (melting/drip) process during a fire. The polymeric nano-additives as such are combined with other traditional flame retardants to produce new flame-retardant materials and materials with favourable mechanical properties. More specifically, a traditional flame retardant can be used in combination with a nano-additive so that the maximum rate of heat release from the material, and indirectly the fire power, is lower than when using the traditional flame retardant alone. As a result, polymer nanocomposite technology, irrespective of the nanofiller, is virtually a universal way to solve the problem of delayed synergistic flame retardants.

Thus far, there is no extensive analysis of the flammable, smoke-generating and toxicity properties of this resin selected for testing—Epidian 5, with the applied additives and mixed modifications. Therefore, the main aim of this work was to solve the flammability problem of epoxy resin by obtaining an effective series of fire-retardant modifications of this material, and then to perform a comprehensive flammability analysis, including the explanation of the mechanism of action of the fire retardants used.

## 2. Materials and Methods

The subject of this study was the preparation of a series of cross-linked, modified epoxy resin materials, and the subsequent analysis of their thermal decomposition products in the combustion process.

### 2.1. Materials

The polymer base was an epoxy resin from the “Organika-Sarzyna” Chemical Plant in Nowa Sarzyna called Epidian 5 (Ep5, a commercial product). It has a broad range of applications, e.g., in construction, electronics, as well as in everyday life. Among other things, it is used in the production of flooring compounds, concrete primers and as a component of adhesives and varnishes. Additionally, it serves as a binder for epoxy-glass laminates and in electronics for flooding electrical and electronic components. It is a viscous liquid characterised, among others, by minimal shrinkage while curing, the possibility of curing at room temperature and good adhesion to most materials, such as metals, glass, ceramics and wood. Additional features include good mechanical properties and resistance to a wide range of chemical agents, such as oils, greases, diluted lyes and acids, resistance to short-term exposure to temperatures up to 80 °C and excellent dielectric properties [30]. Considering the use of the cured material formed of Epidian 5 in public buildings, it is relevant to understand the fire parameters of this material and of its modifications. Table 1 provides the typical parameters of epoxy resin Epidian 5.

Halogen-free mixtures were used, such as organophosphorus ones, i.e., Roflam B7 and Roflam F5 (description below), and nano-additives serving as nanofillers, Mg(OH)_2_ 99% and Al(OH)_3_ 99.9%, whose distributor was Alchem Grupa Sp. z o.o. The grading of the hydroxides used was 10 nm. To cure the mixture, use was made of the hardener Z-1 TECZA (triethylenetetramine), with the chemical formula C_6_H_18_N_4_.

Roflam F5—A phosphoric flame retardant, phenyl-isopropyl-phenyl phosphate, which provides a high flame retardancy profile to plastics. During the combustion process, the product acts in the solid phase, forming a charred layer on the surface of the plastic, inhibiting the spread of the flame. Roflam F5 is a halogen-free flame retardant, and therefore, no toxic gases are released during combustion in the gas phase, such as HCl or HBr [31].

Roflam B7—A tert-butylphenyl phosphate, a halogen-free phosphoric flame retardant in the form of a colourless liquid with low dynamic viscosity. Considering its very good compatibility with many liquid raw materials, it is successfully used in polyurethane plastics, epoxy resins, vinyl ester resins and unsaturated polyester resins. It is a fire-protection agent that belongs to the group of phosphorus aryl esters [32]. According to [9], thermal decomposition and combustion products modified by flame-retardant polymeric materials containing organophosphorus compounds are less toxic than thermal decomposition and combustion products produced by halogen-based flame retardants. Organophosphorus flame retardants in epoxy materials inhibit the combustion process mainly in the solid phase (creating a charring layer on the surface of the polymeric materials).

### 2.2. Sample Preparation

The flame-retardant modifications analysed for the materials formed from Epidian 5 were obtained by adding the individual flame-retardant components in the appropriate concentrations. The halogen-free admixtures Roflam B7 or Roflam F5 in the range of 5–10% by weight, as well as the nanofillers Mg(OH)_2_ or Al(OH)_3_ in the range of 5–10% by weight, were introduced to the epoxy base—Epidian 5—in appropriate amounts, while simultaneously stirring on a mechanical stirrer at a speed of V = 300 rpm. Once all the ingredients had been added, stirring continued for a further 20 min, after which the entire mixture was placed in an ultrasonic bath for 1 h with continuous stirring on a stirrer. In the next step, the uncured mixture was degassed using a mechanical pump. The unmodified, as well as the modified epoxy resin, was cured in a further step with the addition of the hardener Z-1 at a rate of 12 to 100 parts by weight of resin and poured into the moulded parts. The curing process was carried out at room temperature, and the gelation time was approximately 15 min. Initial curing was achieved after about 3 h, with a cure rate of about 80–90% after 24 h, so the samples were then post-cured by placing in an oven for a further 2 h at 120 °C. The composition and determination of the samples produced are shown in Table 2.

### 2.3. Analytical Techniques

#### 2.3.1. Cone Calorimeter Method

The fire characteristics of the analysed epoxy resin modifications were carried out using the cone calorimeter method in accordance with the standard [33]. With this method, it was possible to simultaneously determine the heat release rate (HRR) and smoke during the combustion of materials subjected to a controlled thermal radiation power of 50 kW/m^2^. The applied thermal radiation power simulated phase I of the fire according to the standard curve “fire temperature–combustion time”.

#### 2.3.2. Thermogravimetric Method

A thermal analysis of both unmodified and modified epoxy resin was performed by the dynamic technique according to [34]. On the basis of the thermogravimetric curves obtained with this method, the following parameters have been determined to compare the thermal stability of the tested materials: temperature of the onset of thermal decomposition, temperature of the maximum rate of mass loss of the sample in the first phase of transformation, temperature of the maximum rate of mass loss of the sample in the second phase of transformation, temperature of 50% mass loss, maximum rate of mass loss in the first phase of transformation, maximum rate of mass loss in the second phase of transformation, and mass of the sample after thermal decomposition.

#### 2.3.3. Single-Chamber Test Method

**Figure 1:**
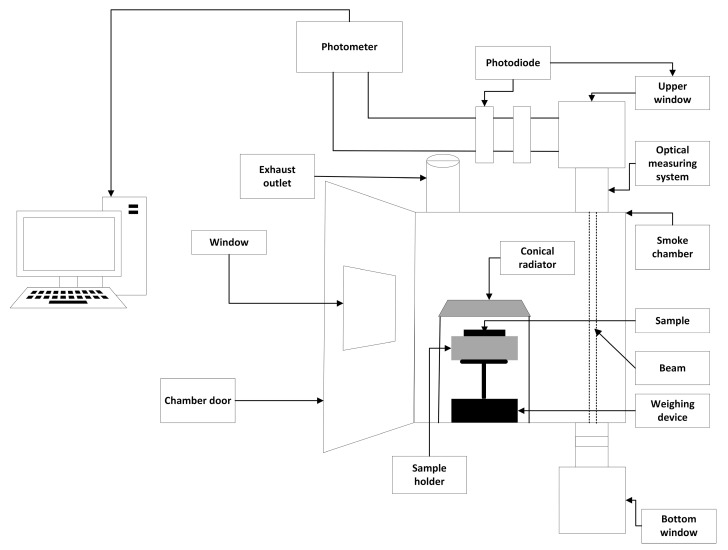
Diagram of a test bed for toxicity testing of thermal decomposition and combustion products according to ISO 5659-2:2017.

In accordance with the standard [35] at a thermal exposure of 50 kW/m^2^, the concentrations of CO_2_ and CO using an FTIR analyser at a combustion time of 600 s were recorded. A schematic of the station is shown in Figure 1.

## 3. Results

### 3.1. Results of Heat Release Rate Analysis of Selected Epoxy Blends

Selected fire parameters of the resulting epoxy modifications are summarised in Table 3.

**Figure 2:**
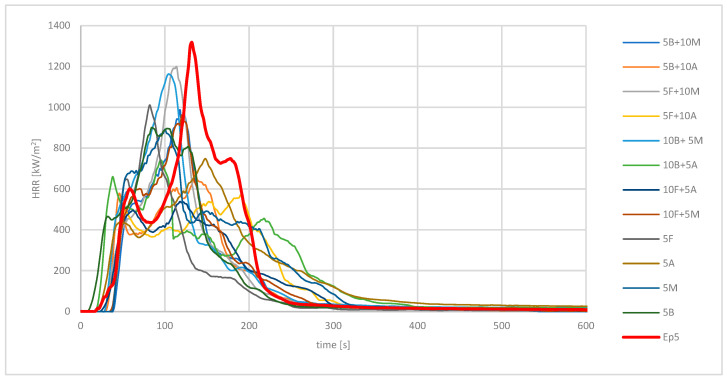
Heat release kinetics of unmodified and modified epoxy material at a heat flux of 50 kW/m^2^ under gas phase ignition conditions.

The heat release rate curves at a thermal exposure of 50 kW/m^2^ of the epoxy materials tested are shown in Figure 2. In the time interval 0 ÷ 16 s, a heating period of the sample was observed, where the absorbed heat emitted from the radiator by the modifications caused their thermal decomposition and the recorded HRR values to be small. Then, for samples 5B and 10B + 5A, there was a significant spike in HRR values (after 10 s). This was due to the presence of phosphate flame retardants, which reduced the thermostability of the polymer material, which formed a layer of char, effectively acting as a flame retardant in the solid phase. On the other hand, a homogeneous modification of 5 wt.% Roflam F5 or a mixed modification of 5 wt.% Roflam B7 and 10 wt.% Mg(OH)_2_ increased the time to ignition of the sample to 38 s.

All the introduced flame retardants were effective at lowering the HRR_max_ and HRR_av_ values of all epoxy resin modifications of Epidian 5 (HRR_max_ by 9 ÷ 59%, HRR_av_ by 1 ÷ 49%). The highest values of HRR_max_ of 1318 kW/m^2^ and HRR_av_ of 603 kW/m^2^ were recorded for Epidian 5. The single-component modification of 5 wt.% Al(OH)_3_ was the most effective at lowering HRR_max_, to 748 kW/m^2^, among the tested resins as a result of water vapour release and the formation of a glassy film during the combustion process, protecting the material from heat and oxygen access. In turn, HRR_av_ was reduced to a value equal to 310 kW/m^2^ after the introduction of 10 wt.% of Roflam B7 and 5 wt.% of Al(OH)_3_. When only one component was used for the modification, the lowest HRR_av_ value of 364 kW/m^2^ was obtained after the introduction of 5 wt.% Mg(OH)_2_. A decrease in SEA_av_ (mean specific extinction coefficient) was observed for all analysed modifications. The lowest value of 158 m^2^/kg (81% lower than Ep5) was recorded for the sample 5M. In contrast, the simultaneous introduction of 5 wt.% Roflam F5 and 10 wt.% Al(OH)_3_ caused the opposite effect and an increase in this parameter. It can be concluded that Roflam F5 accelerated the decomposition of the polymer by forming decomposition products together with aluminium hydroxide, increasing the light attenuation surface area of the smoke particles. After analysing the wt.% of sample residue after combustion, it can be concluded that the effect of the phosphorus-based flame-retardant additives in the combustion of Epidian 5 is mainly related to the gas phase. In the analysed thermal exposure, the introduction of Roflam B7 with aluminium or magnesium hydroxide into the epoxy matrix increased the time to ignition of the sample and the time to reach HRR_max_ for sample 5F + 10A by 20 s and 58 s, respectively. The amount of CO released from 1 kg of modified Epidian 5 material was typically at an average level of 2.3 kg/kg compared to unmodified cured Epidian 5. In contrast, when only hydroxides were used for modification, the amount of CO per 1 kg of material decreased. The amount of CO_2_ released per 1 kg of material, in the case of the 5F + 10A sample, was the lowest, at 10.0 kg/kg (12% lower compared to Ep5). The mechanism of action of the mixed flame retardants was through a synergistic effect. Phosphorus compounds acted as radical scavengers in the gas phase, scavenging free radicals at elevated temperatures in the combustion process, while inorganic compounds, i.e., Mg(OH)_2_ or Al(OH)_3_, caused the release of water vapour, effectively lowering the value of HRR_max_.

### 3.2. Results of Thermogravimetric Analysis

On the basis of the TG and DTG thermogravimetric curves, the values of selected parameters describing the thermal decomposition process of the epoxy materials tested were obtained, which are summarised in Table 4.

Data presented in Table 3 clearly indicate that all fire-retardant modifications of Epidian 5 had a higher percentage of sample residue after thermal decomposition compared to the cured unmodified epoxy resin. Among the single-component additives, the highest residue after thermal decomposition and combustion was recorded for sample 5M, and among the two-component additives for sample 5B + 10A. These residues were five times and almost six times higher, respectively, in relation to Epidian 5. The initial temperatures of thermal decomposition of phase I of cured epoxy resin samples modified with Mg(OH)_2_ (sample 5M) or 5 wt.% organophosphorus compounds and 10 wt.% magnesium hydroxide (samples 5B + 10M and 5F + 10M) were higher compared to cured Epidian 5 (by 7 °C—5M, by 10 °C—5B + 10M, by 5 °C—5F + 10M). The temperatures of the maximum sample weight loss rate in phase I of the transformation were highest for the 5M sample. The difference in values between Ep5 and 5M was 22 °C.

Analysing the epoxy modifications, it can be seen that a synergistic effect was achieved by the addition of 5 wt.% to the epoxy resin Roflam F5 and 5 wt.% Mg(OH)_2_, as the half-life was most effectively lowered compared to the unmodified sample.

### 3.3. An Analysis of Concentrations of the Main Toxic Products Obtained from the Study of the Thermal Decomposition and Combustion Products of the Epoxy Materials Tested

**Figure 3:**
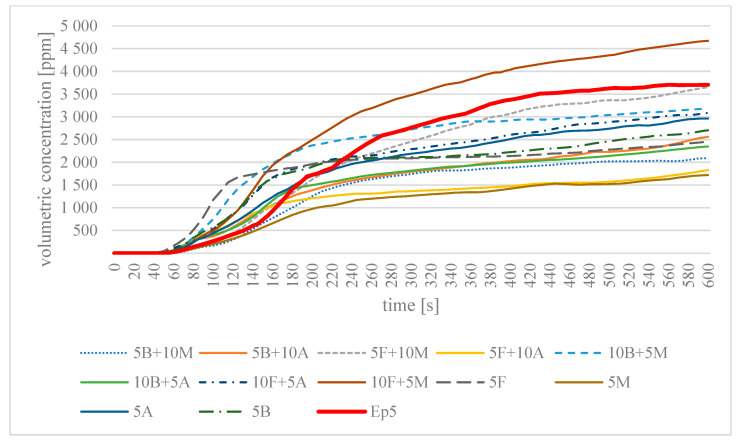
Dependence of the change in the CO volume concentration during combustion of the tested epoxy materials as a function of combustion time in a single-chamber test.

**Figure 4:**
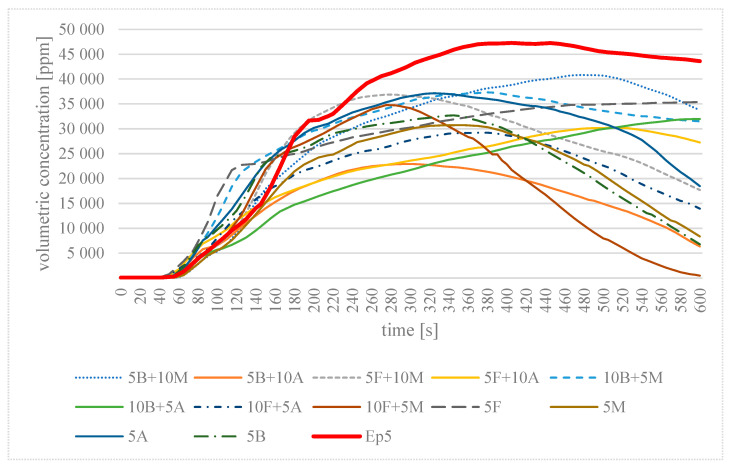
Dependence of the change in the CO_2_ volume concentration during combustion of the tested epoxy materials as a function of combustion time in a single-chamber test.

All analysed samples were found to release CO and CO_2_. Table 5 summarises the results of maximum values of the concentrations of the emitted toxic gases in the process of 10 min of thermal decomposition and combustion of the analysed epoxy modifications, together with the time to reach this value in a closed chamber with a volume of 0.51 m^3^. Figure 3 and Figure 4 present changes in the concentration of the identified chemical compounds, i.e., CO and CO_2_, as a function of time in the process of combustion of flame-retardant epoxy resin modifications.

On the basis of the maximum values obtained for the concentration of the toxic gases emitted, it is clear that for all the samples analysed, the maximum value of the CO concentration was obtained at 600 s, the final time of analysis. The lowest CO value was recorded for sample 5M—at 1721 ppm. In the case of CO_2_ concentration, the time to reach this value was different for each modification. For example, sample 5F obtained the highest value of 35,389 ppm at the end of the analysis time. In contrast, the shortest time of 275 s was characteristic of the 10F + 5M sample, which at the same time had the highest CO value during analysis. For the cured unmodified epoxy resin, the highest CO_2_ concentration value of 47,271 ppm was recorded in the analysed volume of the test chamber.

Figure 3 shows the change in the CO volume concentration during the analysis according to the standard [35]. In the initial phase of sample combustion, a sharp increase in the CO concentration was observed for sample 5F. In the subsequent combustion process, this increase no longer occurred so sharply, and at 600 s, the CO volume concentration for this sample was 2453 ppm. In the case of sample 10F + 5M, it can be seen that the increase in the CO concentration initially slightly increased, and a sharp jump was observed from about 180 s. At 600 s, the CO volumetric concentration in the chamber was 4673 ppm and was the highest of the results of all the samples analysed. Considering the other samples, an analogous increase in the CO volumetric concentration may be observed from about 120 s onwards. At 600 s, concentrations in the range 1721–3704 ppm were recorded. At 600 s of analysis, the materials (5B + 10M, 5B + 10A, 5F + 10M, 5F + 10A, 10B + 5M, 10B + 5A, 10F + 5A, 5F, 5M, 5A, 5B) gave off less CO than the unmodified cured epoxy resin Epidian 5 and the sample 10F + 5M.

From Figure 4 concerning the change in the CO_2_ volumetric concentration during the analysis, the exact time at which the increase in the CO_2_ concentration occurred can be observed, which was also the time at which combustion of the sample began. In the initial phase, the increase in the CO_2_ concentration for most of the samples analysed was almost identical, with the exception of 5M + 10B and the unmodified Epidian 5 sample. In the case of the 10F + 5M sample, by 275 s, the CO_2_ concentration of this sample had risen to a value of 34,783 ppm, and then it gradually decreased, reaching its lowest value of 467 ppm at 600 s. The CO_2_ concentration of the Epidian 5 sample continued to increase for most of the measured time, reaching the highest value of all the samples analysed, equal to 47,271 ppm at 405 s, and then it decreased to a value equal to 43,614 ppm at 600 s. The CO_2_ volumetric concentration values of the other samples analysed up to 600 s ranged from 6346 to 35,367 ppm.

Taking Figure 4 and Table 4 as a basis, it can be concluded that in the tested chamber with a volume of 0.51 m^3^ at a combustion time of 600 s, the maximum concentration of CO_2_ released from the samples of fire-modified Epidian 5 was lower than the corresponding maximum concentration for Epidian 5. This difference—depending on the additive used—ranged from 56,447 to 23,240 ppm.

## 4. Discussion

On the basis of the study, it can be concluded that the introduction of flame-retardant additives consisting of aluminium hydroxide, magnesium hydroxide and organophosphorus compounds into the Epidian 5 epoxy resin reduced its flammability and ignitability, as well as the toxicity of thermal decomposition and combustion products. Increasing the concentration of hydroxides and organophosphorus compounds in the epoxy material tested improved the fire safety. This finding was confirmed by, among other things, a reduction in the maximum rate of heat released from the burning epoxy modification (by 9–59%), which translates into a reduction in the power of the actual fire in which they were burned, similar to that reported in the literature [36,37,38,39,40].

On the basis of the analysed modifications of the epoxy resin, it is possible to confirm the inhibitory effect of the applied flame retardants: magnesium hydroxide, aluminium hydroxide, Roflam B7 and Roflam F5, and their mixtures.

The maximum value of the heat release rate (HRR_max_) and the average value of the heat release rate (HRR_av_) of the modification of Epidian 5 through the introduction of single flame retardants as well as the flame-retardant system were found to be lower than the values of the same parameters for the cured unmodified epoxy material. This demonstrates that the flame retardants used are effective combustion modifiers for epoxy materials obtained on the basis of Epidian 5. The most effective modification of Epidian 5, taking into account the average and maximum heat release rates, was the introduction of 5 wt.% Mg(OH)_2_ in the case of single-component additives or, for mixed compositions, 10 wt.% Roflam F5 and 5 wt.% Al(OH)_3_.

The HRR curves of epoxy materials containing anti-pyrenes/aluminium hydroxide blends had a different shape than the HRR curves of the other tested materials. To a greater extent, a charred layer formed on these modifications. The HRR curves of samples consisting of epoxy resin, Roflam B7 or Roflam F5 and aluminium hydroxide showed a characteristic “saddle”, indicating the formation of a charred layer, which is a barrier to heat and oxygen from the environment, causing inhibition of the combustion process.

The thickest char layer was observed for sample 5F in comparison with other one-component additives introduced into the epoxy matrix. This fact testifies to the fire-retardant effect of the flame retardant used in the solid phase of this modification. In addition, at the analysed thermal exposure, the cured sample of unmodified epoxy resin was practically completely burned. The charred layer was only 1.85% by weight.

The action mechanism of mixed fire retardants was based on the effect of synergism. Phosphorus compounds acted as radical eliminators. They operated in the gas phase and scavenged free radicals at elevated temperatures in the combustion process.

In turn, inorganic compounds (i.e., aluminium (III) hydroxide or magnesium (II) hydroxide) resulted in the evolution of water vapor, which effectively reduced the HRR_max_ value. The obtained results were confirmed by the research team in another publication [41].

The onset temperature of thermal decomposition of compositions mixed simultaneously with hydroxides and organophosphorus compounds is for the most part lower than that of compositions modified with hydroxides alone or with Epidian 5. The simultaneous introduction of 5 wt.% Roflam F5 and 10 wt.% magnesium hydroxide into the epoxy resin most effectively increased the onset temperature of thermal decomposition of phase I (this temperature is 340 °C). The opposite results were obtained when 10 wt.% was used for the modification of Roflam F5 and 5 wt.% for aluminium hydroxide (the temperature value decreased to 310 °C). The mass of ash (%) obtained from the combustion of the cured modified epoxy resin samples during thermogravimetric analysis for most of the epoxy modifications analysed was higher in relation to Epidian 5. The exception was sample 5F (value 3% lower in relation to Epidian 5). Sample 5M was characterised by the highest value of ash mass (%) in relation to the single-component modifications, equal to 7.30%. Among the samples consisting of two additives, materials containing 5 wt.%. Roflam B7 + 10 wt.% Al(OH)_3_ showed a higher susceptibility to form a charred layer on the surface of the epoxy resin compared to the cured unmodified epoxy resin and the other two-component modifications. Flame retardants containing a phosphorus atom or atoms in the particular polymer arrangements usually belong to the ecological anti-pyrenes [42]. Anti-pyrenes that contain phosphorus are effective as flame retardants in the epoxides because the epoxides contain many oxygen atoms (similar to polyesters, polyurethanes or cellulose). While comparing epoxy materials without phosphorus compounds with fire-resistant anti-pyrenes containing phosphorus compounds, one can notice that modified epoxy materials are usually less thermally stable and have lower starting temperatures of thermal decomposition, but exhibit multilevel thermal decomposition with higher efficiency of the carbonised residue [43,44].

On the basis of the toxicity analysis of all the tested samples, CO and CO_2_ were released. The CO concentration value increased over the test time of 600 s. The lowest CO value of 1721 ppm was recorded for sample 5M. This was 54% lower than Epidian 5. At the same time, the same sample had the lowest CO_2_ value of 30,751 ppm. After analysing the CO_2_ concentration, the time to the highest value for each modification differed. For example, sample 5F obtained the highest CO_2_ value of 35,389 ppm at the end of the analysis time. In contrast, the fastest maximum CO_2_ value, at 275 s, was recorded for the 10F + 5M sample, for which, at the same time, the CO value at the time of analysis was the highest. The lowest CO_2_ value of 22,931 ppm was characterised by sample 5B + 10A, while the highest CO_2_ value of 47,271 ppm was recorded for the cured unmodified epoxy resin.

## 5. Conclusions

From the research carried out and from the literature review, it was concluded that:

- All the applied fire-retardant additives to the epoxy resin Epidian 5 effectively changed the fire properties of the epoxy materials tested. The heat release rate was reduced compared to the non-fire-modified material. The HRR_max_ and HRR_av_ values of all fire-retardant modifications were lower compared to the corresponding HRR values of the unmodified Epidian 5 material (HRR_max_ lower by 9–59%, HRR_av_ lower by 1–49%).
- The solid-phase inhibitory effect of the applied flame retardants and their mixtures has been confirmed by the formation of layers of char.
- Applied anti-pyrenes containing phosphorus were found to be less thermally stable and have lower starting temperatures of thermal decomposition, but they exhibited multilevel thermal decomposition with higher efficiency of the carbonised residue.
- For samples 5F and 5B + 10M, the highest value of time to ignition of the gas phase was obtained (38 s). The main action of the additives took place in the solid phase. This was also evidenced by the higher residue values after thermal decomposition and combustion (by 76–790%) compared to the unmodified sample.
- The blended compositions had a lower onset temperature of thermal decomposition of the first phase compared to Epidian 5.
- The introduction of 5 wt.% magnesium hydroxide into Epidian 5 increased the initial temperature of thermal decomposition of phase I by 7 °C.
- Majority of the fire-retardant modifications of the epoxy resin were characterised by a higher ash weight (%) in relation to Epidian 5. The exception was sample 5F (3% lower value in relation to Epidian 5).
- The introduction of 5 wt.% magnesium hydroxide caused an increase in ash weight (%) in the thermogravimetric analysis to the highest differential level of 7.30%, as compared to all single-component flame-retardant modifications.
- CO and CO_2_ were identified in the toxic gases included in the smoke from the combustion of the samples analysed, according to the research methodology selected for the study.
- The concentration of CO (ppm) in thermal decomposition and combustion increased throughout the analysis lasting 600 s.

## Figures and Tables

**Table 1:** Typical parameters of epoxy resin based on Epidian 5 [30].

Parameter	Characteristic Features
Melting point/freezing point	30–50 °C
Glass transition temperature	−16 °C
Ignition temperature	266 °C
Vapour pressure (20 °C, 50 °C)	4.6 × 10^−8^ Pa
Density (20 °C)	1.16 g/cm^3^
Viscosity (25 °C)	20,000–30,000 mPas

**Table 2:** Composition and determination of the analysed samples.

Item	Sample Name	Sample Composition
1.	Ep5	Ep5
2.	5B	Ep5 + 5 wt.% Roflam B7
3.	5A	Ep5 + 5 wt.% Al(OH)_3_
4.	5M	Ep5 + 5 wt.% Mg(OH)_2_
5.	5F	Ep5 + 5 wt.% Roflam F5
6.	10F + 5M	Ep5 + 10 wt.% Roflam F5 + 5 wt.% Mg(OH)_2_
7.	10F + 5A	Ep5 + 10 wt.% Roflam F5 + 5 wt.% Al(OH)_3_
8.	10B + 5M	Ep5 + 10 wt.% Roflam B7 + 5 wt.% Mg(OH)_2_
9.	10B + 5A	Ep5 + 10 wt.% Roflam B7 + 5 wt.% Al(OH)_3_
10.	5F + 10M	Ep5 + 5 wt.% Roflam F5 + 10 wt.% Mg(OH)_2_
11.	5F + 10A	Ep5 + 5 wt.% Roflam F5 + 10 wt.% Al(OH)_3_
12.	5B + 10M	Ep5 + 5 wt.% Roflam B7 + 10 wt.% Mg(OH)_2_
13.	5B + 10A	Ep5 + 5 wt.% Roflam B7 + 10 wt.% Al(OH)_3_

**Table 3:** Thermokinetic and thermophysical properties of cured epoxy resin Epidian 5, modified and unmodified with Al(OH)_3_ or Mg(OH)_2_ and organophosphate flame retardants in ignition conditions at an external heat flux density of 50 kW/m^2^.

Item	Sample Name	HRR_max_ (kW/m^2^)	HRR_av_ (kW/m^2^)	THR * (MJ/m^2^)	SEA_av_ (m^2^/kg)	TSP ** (m^2^)	Time Until Ignition (s)	Time Until Reaching HRR_max_ (s)	Sample Remnants (wt.%)	Proper Emission of CO (kg/kg)	Proper Emission of CO_2_ (kg/kg)
1.	Ep5	1318	603	130	849	40.7	16	132	1.85	2.1	11.3
2.	5B	900	595	114	316	21.3	10	84	3.87	2.2	10.4
3.	5A	748	435	136	197	17.0	16	148	5.64	1.9	10.5
4.	5M	892	364	117	158	15.6	36	100	3.25	2.0	10.6
5.	5F	1011	446	77	655	33.6	38	82	5.74	2.9	10.6
6.	10F + 5M	962	475	101	701	35.3	34	120	7.60	2.4	10.4
7.	10F + 5A	539	353	84	693	35.0	16	118	16.47	2.3	10.1
8.	10B + 5M	1162	584	121	476	27.1	30	104	5.57	2.2	11.6
9.	10B + 5A	740	310	105	327	21.7	10	96	10.22	2.3	10.5
10.	5F + 10M	1199	530	107	278	19.9	10	114	6.97	2.2	11.6
11.	5F + 10A	570	369	104	926	43.5	36	190	9.28	2.1	10.0
12.	5B + 10M	988	470	96	754	37.2	38	118	11.01	2.4	11.9
13.	5B + 10A	693	405	87	547	29.7	30	136	9.80	2.7	11.3

* THR (total heat release), ** TSP (total smoke production).

**Table 4:** Thermogravimetric test results of cured modified and unmodified epoxy resin samples at a heating rate of 5 °C/min.

Item	Sample name	Temperature of Onset of Thermal Decomposition of the First Transformation Phase (°C)	Temperature of the Maximum Rate of Weight Loss of the Sample in Phase I/II of the Transformation (°C)	Maximum Rate of Mass Loss in Phase I/II Transformation (%/min)	Temperature of 50% Sample Weight Loss (°C)	Mass of Sample after Thermal Decomposition (mg); (%)
1.	Ep5	330	343/514	6.71/2.99	395	0.05; 1.56
2.	5B	317	335/510	6.70/2.36	368	0.14; 4.44
3.	5A	335	337/511	9.77/2.50	399	0.19; 5.31
4.	5M	337	365/487	6.99/21.36	376	0.25; 7.30
5.	5F	318	330/512	7.53/1.99	361	0.06; 1.52
6.	10F + 5M	316	338/512	7.18/2.53	380	0.19; 5.59
7.	10F + 5A	310	333/519	5.99/2.01	360	0.22; 6.71
8.	10B + 5M	320	338/522	7.46/2.06	374	0.22; 5.82
9.	10B + 5A	311	328/513	7.50/1.97	355	0.27; 6.99
10.	5F + 10M	335	346/510	6.85/3.38	346	0.27; 7.02
11.	5F + 10A	320	336/513	7.61/2.15	366	0.26; 7.00
12.	5B + 10M	340	353/508	7.41/3.08	388	0.27; 7.85
13.	5B + 10A	319	337/515	6.94/2.13	372	0.34; 8.77

**Table 5:** Summary of the maximum concentrations of toxic gases released during the combustion process for the analysed epoxy modifications.

Item	Sample Name	Maximum CO Concentration (ppm)	Maximum CO_2_ Concentration (ppm)	Time to Reach Maximum Concentration Value (s)
1.	Ep5	3705	47,271	405
2.	5M	1721	30,751	355
3.	5A	2963	37,170	325
4.	5B	2707	32,725	345
5.	5F	2453	35,389	600
6.	5B + 10A	2561	22,931	300
7.	5B + 10M	2091	40,824	475
8.	10B + 5A	2348	31,989	595
9.	10B + 5M	3183	37,338	380
10.	5F + 10A	1830	30,212	510
11.	5F + 10M	3656	36,875	285
12.	10F + 5A	3083	29,224	380
13.	10F + 5M	4673	34,783	275

## Data Availability

The data are provided in the article.
